# Prosthodontic Rehabilitation of a Patient With Psoriasis and Severe Medication-Related Nausea Using Telescopic Crown-Retained Removable Prostheses: A Clinical Report

**DOI:** 10.7759/cureus.111542

**Published:** 2026-06-26

**Authors:** Panagiota Chatzidou, Athanasios Stratos, Athanasios Tsekos, Vasilios Stefos, John Fanourgiakis

**Affiliations:** 1 Dentistry, Private Practice, Thessaloniki, GRC; 2 Prosthodontics, School of Dentistry, Faculty of Health Sciences, Aristotle University of Thessaloniki, Thessaloniki, GRC; 3 Management Science and Technology, Hellenic Mediterranean University‎, Heraklion, GRC

**Keywords:** computer-aided design/computer-aided manufacturing (cad/cam), digital dentistry, methotrexate therapy, nausea management, oral rehabilitation, psoriasis, removable dental prosthesis, telescopic crowns

## Abstract

The rehabilitation of partially edentulous patients with complex medical history presents substantial clinical challenges, particularly when both implant-supported and conventional removable prosthetic options are limited. This case report describes the comprehensive management of a 67-year-old female patient referred for prosthetic rehabilitation of a severely compromised dentition. The patient expressed a strong preference for implant-supported restorations; however, due to her autoimmune condition (psoriasis) and ongoing methotrexate therapy, implant placement was contraindicated. Additionally, persistent vomiting and severe nausea significantly complicated the clinical procedure.

A patient-centered and interdisciplinary approach was adopted. In collaboration with the patient’s physician, methotrexate was temporarily discontinued to improve tolerance to dental treatment. Following extractions of non-restorable teeth, a telescopic crown-retained removable complete denture (RCD) was selected as the definitive treatment plan for both dental arches. Digital workflows were implemented with intraoral scanning, and both the mandibular and maxillary arches were restored.

At 18-month follow-up, the patient demonstrated excellent functional and aesthetic outcomes, with complete resolution of nausea symptoms during prosthesis use. This case highlights the importance of individualized treatment planning, digital prosthodontic workflows, and the advantages of telescopic systems in managing medically compromised patients with high aesthetic demands and functional limitations.

## Introduction

The restoration of partially edentulous patients requires careful consideration of biological, mechanical, and patient-related factors. While implant-supported prostheses are widely regarded as the gold standard for oral rehabilitation, their application is not always feasible due to systemic contraindications, medication-related risks, or patient-specific limitations [1].

Autoimmune diseases, such as psoriasis, are frequently managed with systemic immunomodulatory agents, including methotrexate [2]. Methotrexate is a folate antagonist that inhibits DNA synthesis and cellular proliferation, making it effective in controlling inflammatory conditions. However, it is associated with numerous adverse effects relevant to dental care, including mucositis, immunosuppression, delayed wound healing, hepatotoxicity, and gastrointestinal disturbances such as nausea and persistent vomiting. These effects complicate both surgical and prosthetic dental interventions [3].

In addition to systemic factors, functional limitations such as exaggerated nausea can significantly impair dental treatment. Nausea interferes with routine dental procedures, including conventional impression making and even intraoral scanning in digital workflows. Management requires a combination of behavioral, technical, and sometimes pharmacological strategies.

Telescopic crown systems represent a valuable alternative in complex prosthodontic cases. These systems consist of primary copings cemented onto prepared abutment teeth and secondary frameworks incorporated into a removable prosthesis. Their advantages include superior retention, favorable load distribution, improved aesthetics due to the absence of visible clasps, and retrievability for maintenance and dental hygiene [4].

Recent advancements in digital dentistry have significantly influenced the fabrication of telescopic prostheses. Computer-aided design and computer-aided manufacturing (CAD/CAM) technologies enable precise control of taper angles, improved fit, and reproducible retention forces compared to conventional analog techniques [5]. Digital workflows also allow the use of high-performance materials such as zirconia and PEEK, which demonstrate favorable mechanical and retentive properties [6, 7]. Intraoral scanning is recommended, particularly in cases with clinical limitations such as severe nausea, where analog techniques may be challenging and modified conventional approaches may be required [8].

This case report presents the staged rehabilitation of a medically compromised patient with severe nausea, using mandibular and maxillary removable complete dentures retained by telescopic crowns, integrating digital and analog workflows, interdisciplinary collaboration, and adaptive clinical strategies.

## Case presentation

**Figure 1:**
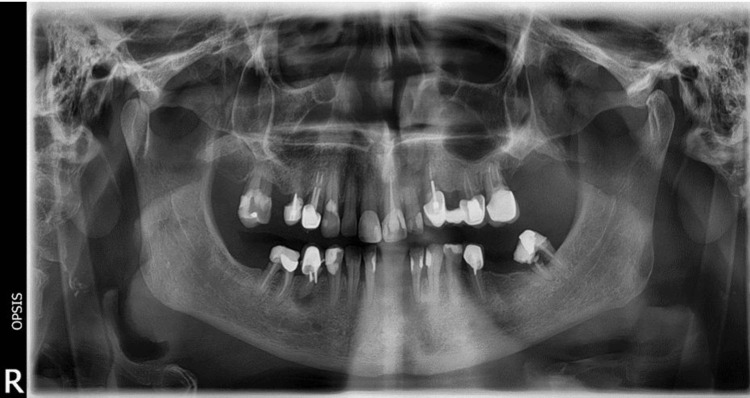
Preoperative Orthopantomograph

**Figure 2:**
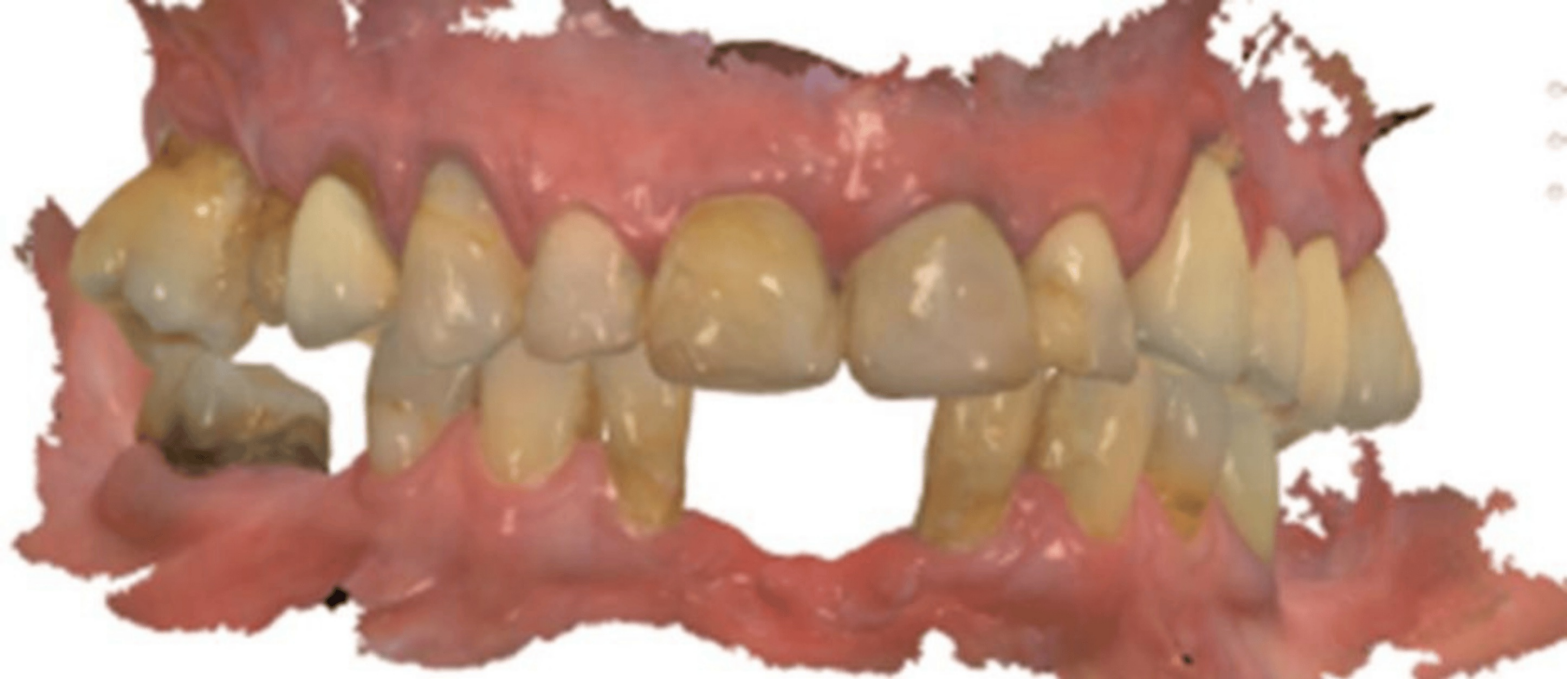
Preoperative Intraoral Scanning

A 67-year-old female patient was referred by a maxillofacial surgeon for prosthodontic rehabilitation. The patient presented with a severely compromised dentition, confirmed clinically and radiographically via a panoramic radiograph (orthopantomogram) (Newtom GO 2D/3D, CEFLA S.C., Italy) (Figure 1). Multiple teeth were deemed non-restorable due to extensive caries, periodontal disease, and structural compromise (Figure 2).

The patient expressed a strong desire for implant-supported rehabilitation due to aesthetic concerns. However, her medical history was significant for psoriasis, managed with methotrexate therapy. Given the immunosuppressive effects of methotrexate and the associated risks of impaired osseointegration and postoperative complications, implant therapy was contraindicated.

A further complicating factor was the presence of persistent vomiting and pronounced nausea. Even routine intraoral examination triggered gagging episodes, significantly limiting clinical procedures and necessitating modifications to standard protocols.

Following detailed consultations and informed consent, a treatment plan was developed prioritizing aesthetics, function, and medical safety. In coordination with the patient’s physician, methotrexate therapy was temporarily discontinued during the active treatment phase to reduce gastrointestinal symptoms and improve procedural tolerance. Table 1 compares the integration of digital and analog protocols in telescopic crown-retained prostheses for the patient with medication-induced nausea. 

**Table 1:** Comparative Integration of Digital and Analog Protocols in Telescopic Crown-Retained Prostheses for Patients With Severe Nausea CAD/CAM: computer-aided design and computer-aided manufacturing; PEEK: polyetheretherketone; PEKK: polyetherketoneketone

Parameter	Digital Workflow	Analog Workflow	Integrated Clinical Perspective
Primary Data Acquisition	Intraoral scanning minimizes impression material use and may enhance patient tolerance; however, it remains technique-sensitive in patients with nausea [9]	Conventional impressions allow greater procedural control and adaptability in compromised patients [8]	A selective approach is recommended, initiating with digital acquisition when feasible and transitioning to modified analog techniques
Accuracy and Marginal Fit	CAD/CAM fabrication ensures high precision and standardized internal fit [5]	Depending on laboratory expertise, variability may occur due to manual processing [5]	Digital design combined with clinical analog verification enhances overall prosthetic accuracy
Retention and Taper Control	Precise control of taper angles and surface characteristics results in predictable retention forces	Retention is influenced by manual fabrication and may be inconsistent	Hybrid workflows enable digitally optimized primary copings with clinically adjusted secondary components
Material Selection	Enables utilization of zirconia, PEEK, and PEKK with favorable biomechanical and aesthetic properties [7]	Primarily limited to metal alloys with proven longevity but inferior aesthetics [10]	Combining ceramic primary crowns with polymer or metal secondary structures optimizes performance and aesthetics
Management of Nausea	Reduced bulk and shorter clinical procedures may decrease nausea triggers, though scanning may still be challenging in severe cases	Allows procedural modifications (e.g., sectional impressions, patient positioning) to better manage nausea [8]	Analog techniques remain indispensable, with digital methods applied selectively to reduce patient discomfort
Clinical Efficiency	Streamlined workflow reduces chairside time and number of appointments [9]	Often requires multiple visits and chairside adjustments	Integration of digital planning with analog execution improves efficiency while maintaining flexibility
Laboratory Workflow	Automated fabrication enhances consistency and reduces human error [5].	Relies on technician skill and manual processes	Digital frameworks can be complemented by analog finishing for individualized adaptation
Reproducibility and Maintenance	Digital data storage enables reproducibility and facilitates long-term maintenance [9]	Reproduction of prostheses is challenging due to lack of standardized records	Hybrid documentation (digital + analog) ensures optimal long-term follow-up
Adaptability to Clinical Limitations	Limited by intraoral conditions such as excessive salivation, restricted access, or severe nausea	Limited adaptability to severe nausea	A hybrid approach allows real-time modification of workflow based on patient tolerance
Long-Term Clinical Outcomes	Demonstrates favorable retention and mechanical stability with modern materials	Proven long-term success and survival in clinical studies [11].	Both approaches are clinically valid; integration enhances predictability and longevity
Overall Clinical Applicability	Highly effective in controlled clinical environments with adequate patient cooperation	Reliable across a wider range of clinical conditions	Integration of digital precision with analog adaptability represents the most pragmatic and patient-centered strategy

Following the extraction of non-restorable teeth (teeth extracted 16, 15, 14, 23, 25, 27, 45, 42, 32, 37) and appropriate healing, prosthodontic rehabilitation commenced with the mandibular arch in order to facilitate the patient’s gradual acceptance and adaptation to the dental treatment process.

Mandibular rehabilitation

**Figure 3:**
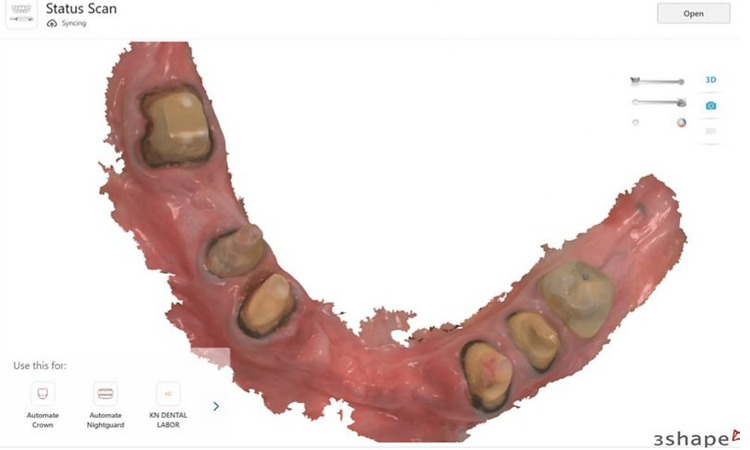
Preparation of Mandibular Abutments; Gingival Retraction; Digital Impressions With Trios 3 Trios 3 (3Shape, Copenhagen, Denmark)

**Figure 4:**
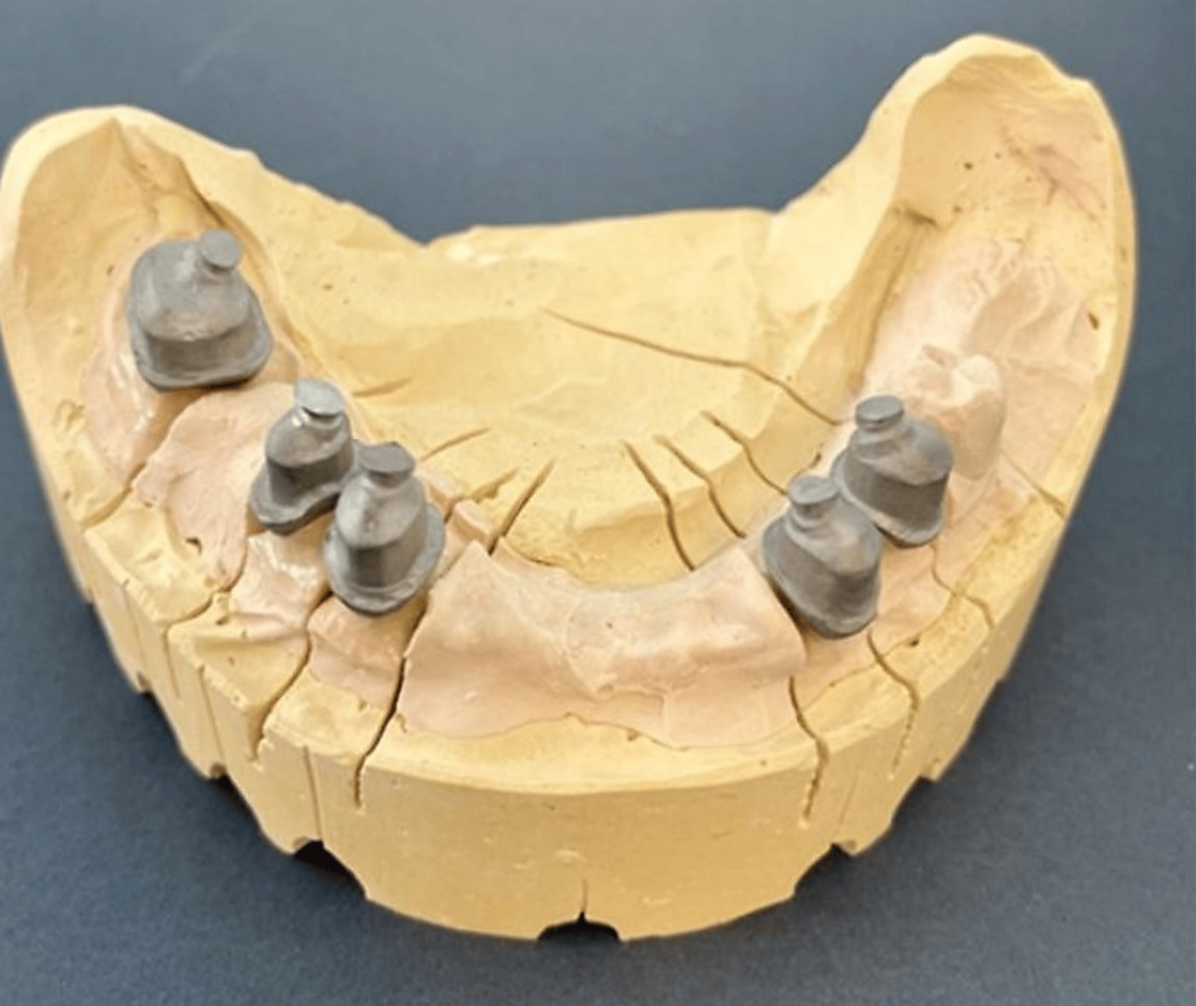
Fabrication and Try-in of Primary Copings

The prostheses were retained using rigid telescopic crowns consisting of CAD/CAM-fabricated parallel-sided primary copings and a cobalt-chromium secondary framework. The primary copings were designed with a 0° taper (parallel walls) to maximize frictional retention and enhance prosthesis stability. The copings extended coronally to provide an adequate frictional surface area while maintaining favorable crown-to-root ratios and periodontal support. Parallelism of all abutments was verified clinically and during laboratory procedures to ensure a common path of insertion. The remaining mandibular teeth were prepared to receive the primary telescopic copings. Gingival retraction was achieved using retraction cords (Ultrapack, Ultradent, South Jordan, UT), and digital impressions were obtained using an intraoral scanner (Trios 3, 3Shape, Copenhagen, Denmark), minimizing patient discomfort and reducing nausea stimulation (Figures 3-4).

Primary copings were fabricated and verified intraorally for fit and parallelism. Border molding was performed using a custom tray (Custom Impression Tray Material ALMORE, Hickory, NC) fabricated from an initial alginate impression (Kerr, USA) and greenstick compound (Kerr, USA), followed by a polyether pickup impression (Polyether Impregum, 3M ESPE, Maplewood, MN) to accurately capture the relationship between the copings and the soft tissues.

**Figure 5:**
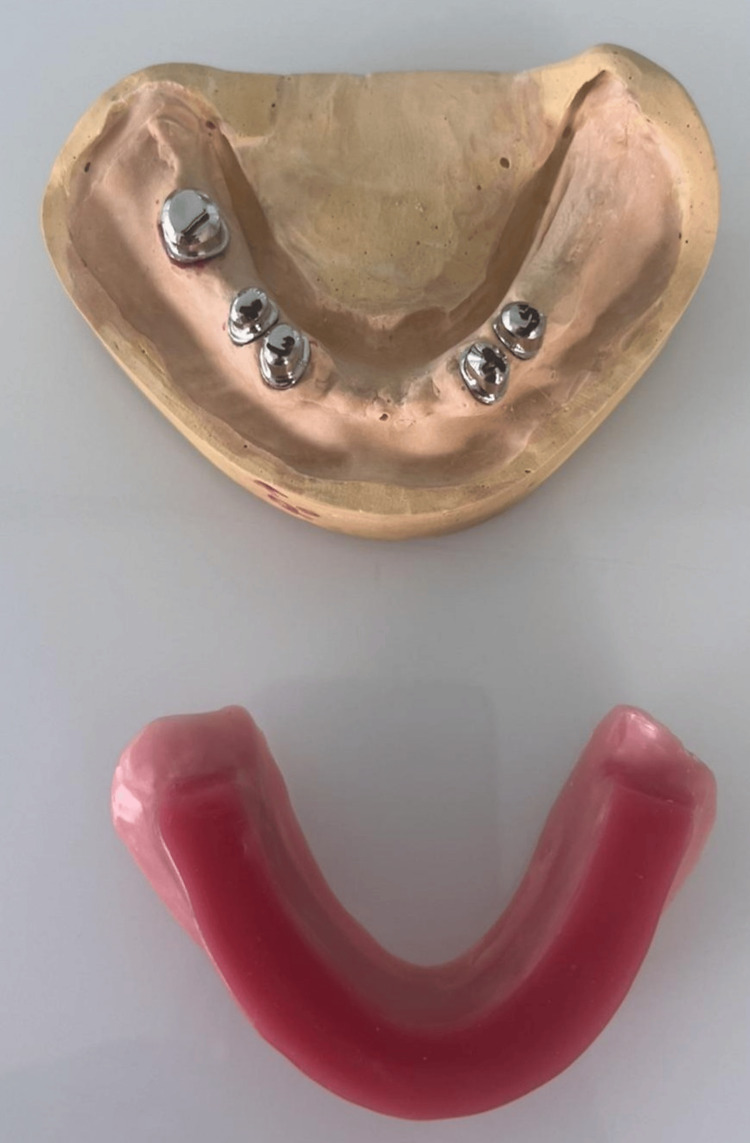
Occlusal Rims; Centric Jaw Relation; OVD Established OVD: occlusal vertical dimension

A cobalt-chromium secondary framework was fabricated (Vitalium 2000 Dentsply Sirona, Charlotte, NC) and tried intraorally. Occlusal rims were constructed (Wax rims / Custom Impression Tray Material ALMORE BMS modeling wax/ALMORE), and the centric jaw relation was recorded at the appropriate occlusal vertical dimension (Figure 5).

Occlusal records were obtained at the established occlusal vertical dimension, and a bilateral balanced occlusal scheme was developed to enhance denture stability during functional and parafunctional movements. The occlusal arrangement aimed to distribute occlusal loads evenly across the abutment teeth and residual ridges, minimizing destabilizing forces and improving patient comfort.

The combination of frictional retention from the telescopic copings, splinting of the abutment teeth, broad stress distribution, and balanced occlusion contributed significantly to prosthesis retention, support, and stability.

**Figure 6:**
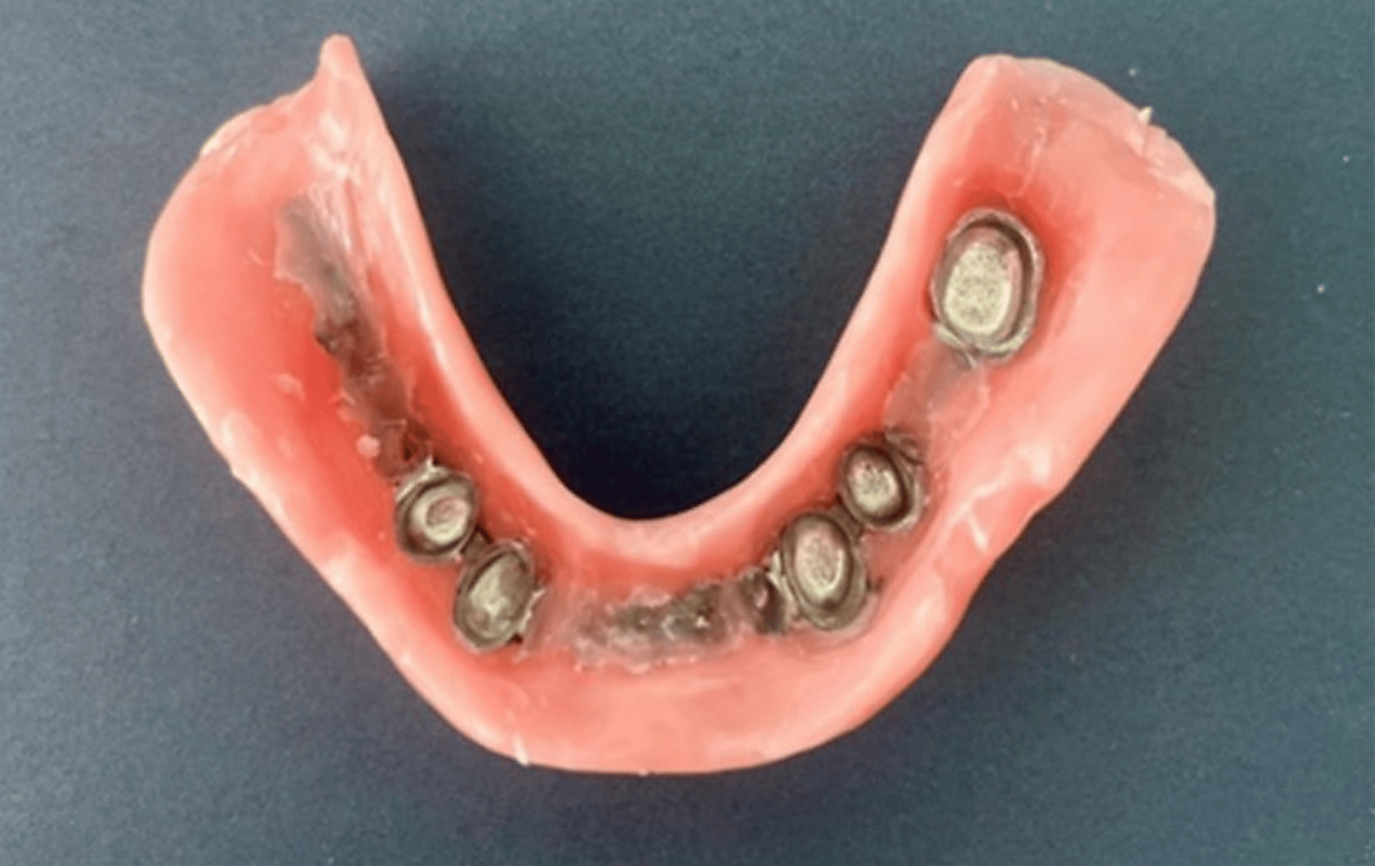
Inside View of Lower Denture

Denture teeth (Phonares Ivoclar Vivadent, Schaan, Liechtenstein) were arranged, and a try-in was performed with the maxillary natural dentition still present, allowing evaluation of phonetics, occlusion, and aesthetics. At that appointment, the second left premolar (35), which was heavily restored with an inadequate post and crown, was reduced to gingival level to a dome shape due to its low restorability index [11]. The final prosthesis was processed using the Ivobase injection molding system (Ivobase injection system, Ivoclar Vivadent) (Figure 6).

At delivery, the primary copings were cemented with glass ionomer cement (Ketac Cem, 3M) simultaneously with the seating of the secondary framework and prosthesis while the patient occluded in maximum intercuspation.

Maxillary rehabilitation

Following successful adaptation to the mandibular prosthesis, attention was directed to the maxillary arch. Due to the patient’s severe nausea, a staged approach was implemented.

**Figure 7:**
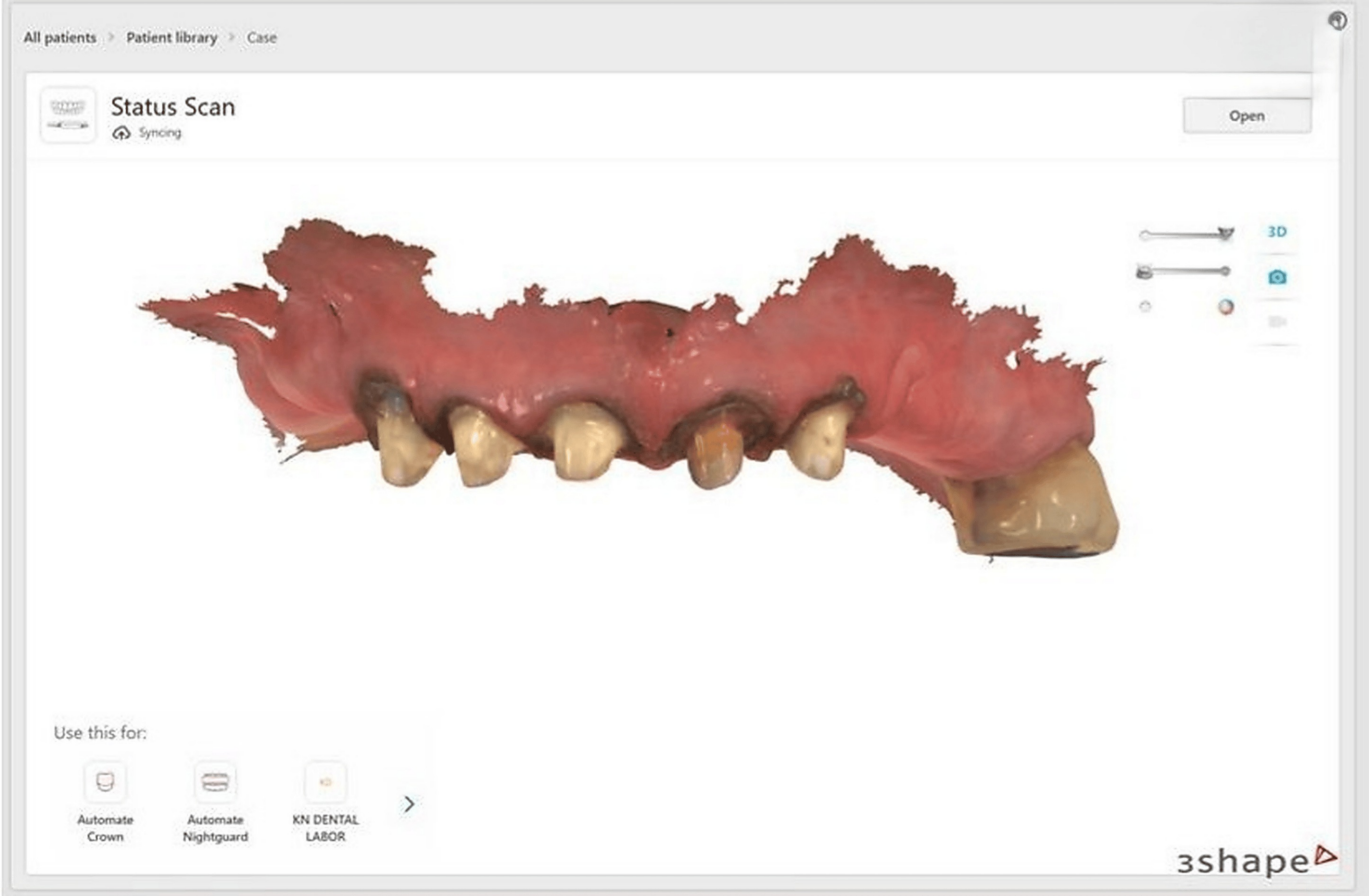
Intraoral Scan of Tooth Preparation

An intraoral scan of the maxillary arch was obtained using the Trios 3 system (3Shape) (Figure 7). A provisional partial denture with minimal palatal coverage and clasps on natural teeth was fabricated. This allowed the identification of functional borders and patient tolerance limits, particularly with respect to palatal extension.

The prosthesis was progressively adjusted to achieve optimal comfort, minimizing nausea stimulation while maintaining functionality. This stage was critical in defining the design parameters for the definitive prosthesis.

**Figure 8:**
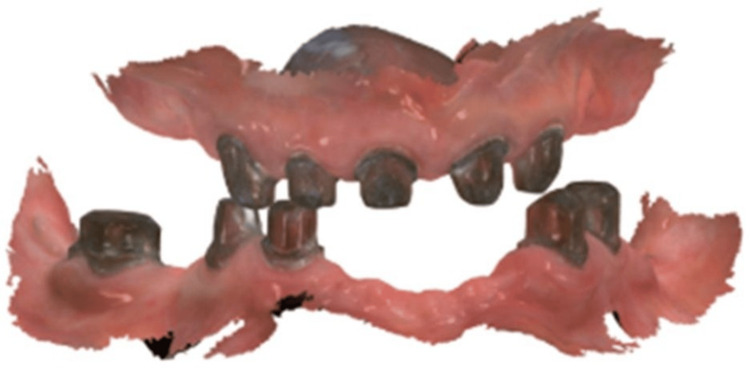
Intraoral Scanning of the Telescopic Crowns In Situ

**Figure 9:**
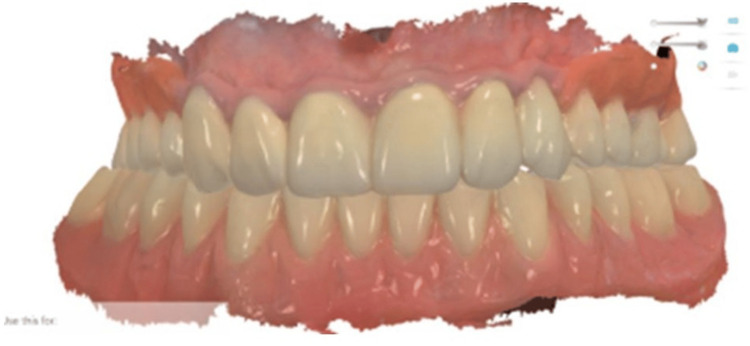
Digital Smile Design

**Figure 10:**
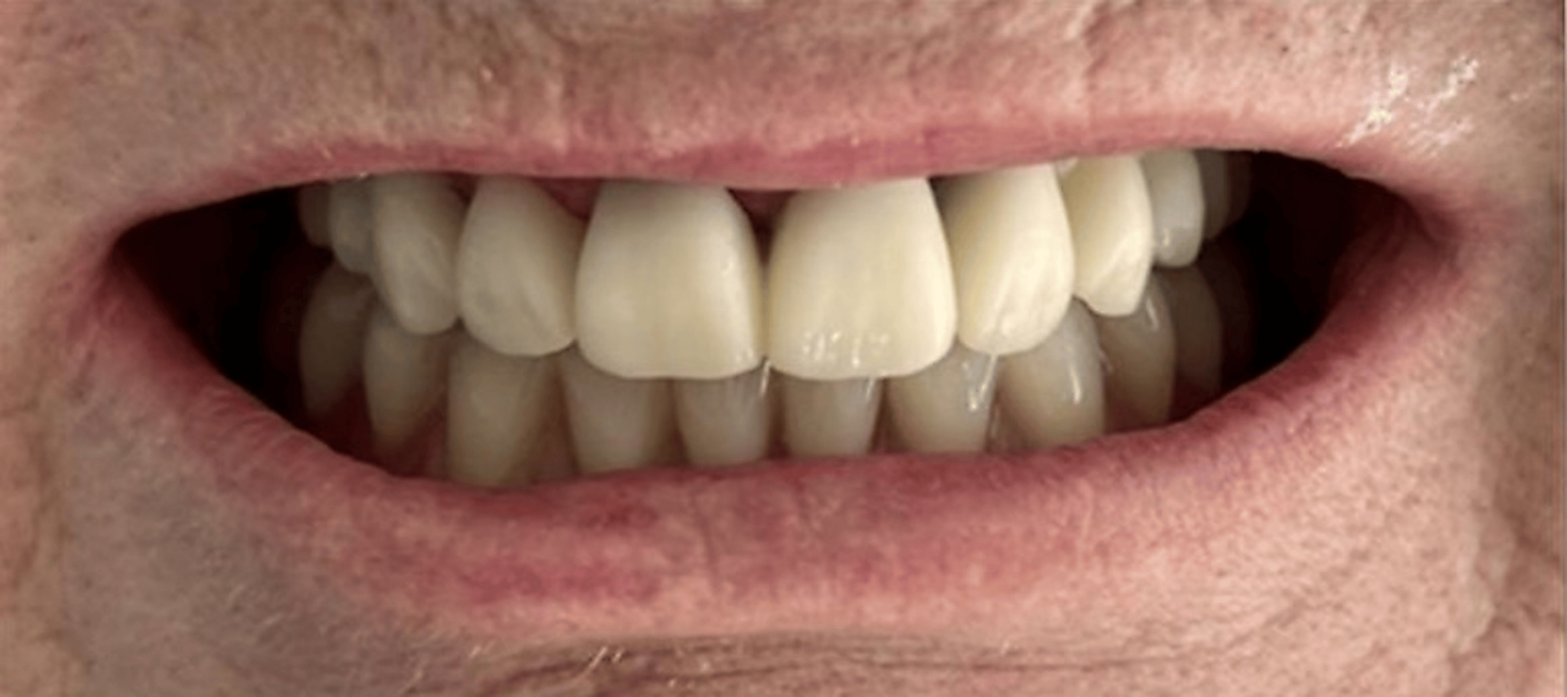
PMMA Try-In of the Upper Denture PMMA: polymethyl methacrylate

Subsequently, the remaining maxillary teeth were prepared for telescopic copings (Figure 8). A fully digital workflow was employed, including digital smile design and CAD/CAM fabrication of primary and secondary frameworks (DentalCAD 3.2 Elefsina, exocad GmbH, Darmstadt, Germany) (Figures 8-9). A PMMA mock-up (Telio CAD, Ivoclar) was used during the try-in phase to evaluate aesthetics and function. and tried intraorally (Figure 10).

**Figure 11:**
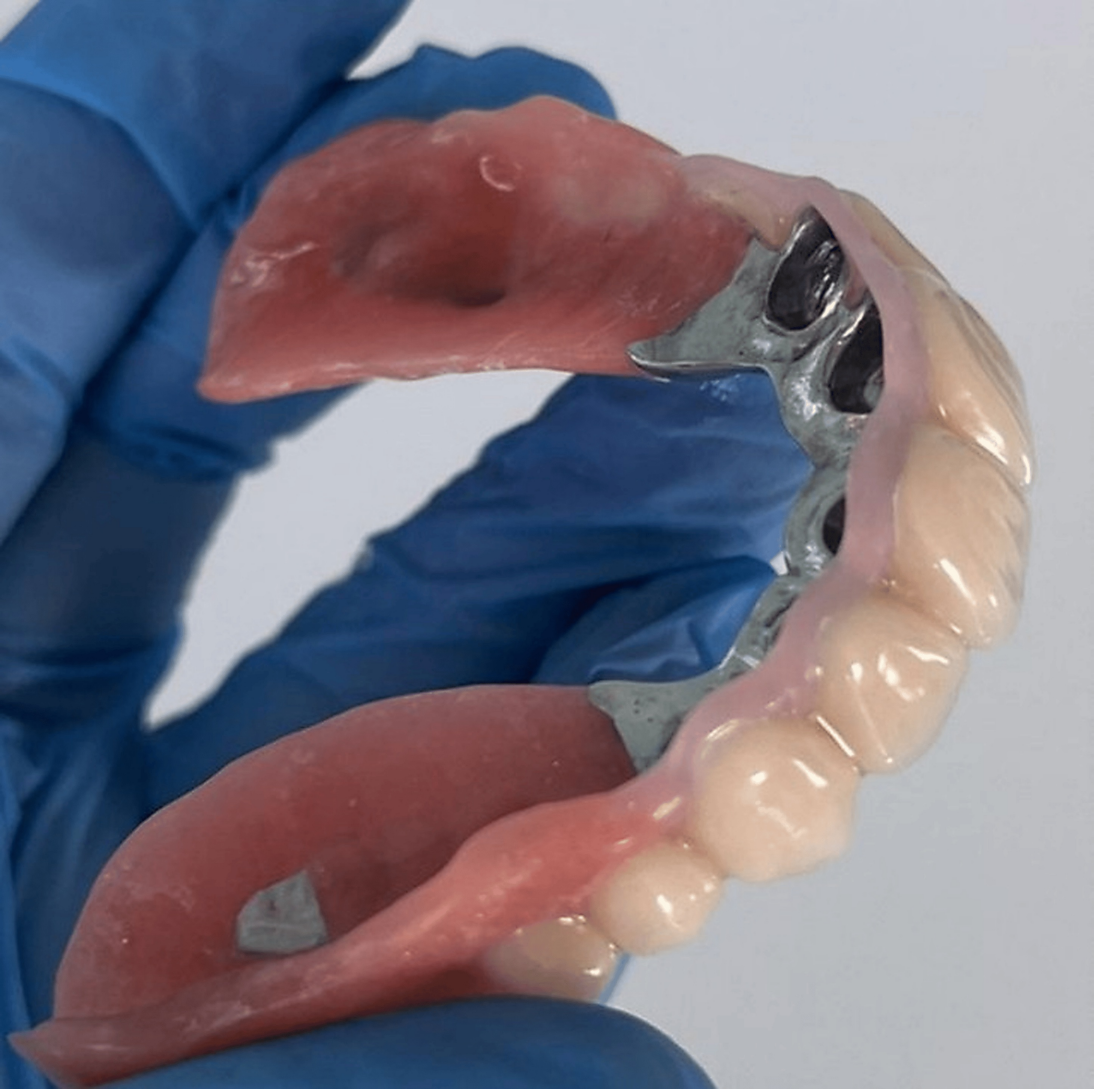
Finished Upper Denture Inside View

The final prosthesis was fabricated using a digital denture manufacturing technology for the acrylic components (Ivotion, Ivoclar Vivadent), which combines pre-polymerized multilayer polymethyl methacrylate (PMMA), anatomical shell geometry, CAD/CAM nesting, and monolithic milling to produce a single-piece engineered denture with enhanced precision, strength, and fit compared with conventional layered fabrication methods. The cobalt-chromium framework (Cobalt-chromium alloy framework Vitalium 2000, Dentsply Sirona) was selected based on patient preference, given concerns about the long-term performance of zirconia-polymer combinations and a desire to avoid retreatment (Figure 11).

At delivery, the primary copings were cemented to the secondary framework, which was integrated within the acrylic RCD, using glass ionomer cement in maximal intercuspation.

Post-delivery complications and management

During the first 48 hours to 1 week following delivery, multiple instances of decementation of primary copings were observed. This was attributed to excessive frictional forces resulting from highly precise, 0° parallelism of the digitally fabricated frameworks.

Management involved recementation of copings and chairside adjustment of the internal surfaces of the secondary framework using rubber burs (Dedeco Rubber Polishers, Dedeco International, Long Eddy, NY) to slightly reduce friction. Additionally, physiological adaptation of the periodontal ligament and functional splinting of abutment teeth contributed to stabilization.

Following these interventions, no further decementation events were reported.

Follow-up and timeline of treatment

The patient was monitored at 1 day, 3 days, 7 days, 2 weeks, 1 month, 6 months, 12 months, and 18 months. At 6 and 12 months, chairside relining using a soft reline material (Quick Up, VOCO, Germany) was performed to compensate for ridge resorption at extraction sites. It was later replaced with a hard laboratory reline.

Adjunctive chemical plaque control measures were recommended, including the use of a 0.12% chlorhexidine mouthrinse for one week each month and daily toothbrushing with sodium fluoride toothpaste to enhance plaque control and minimize the risk of caries.

**Figure 12:**
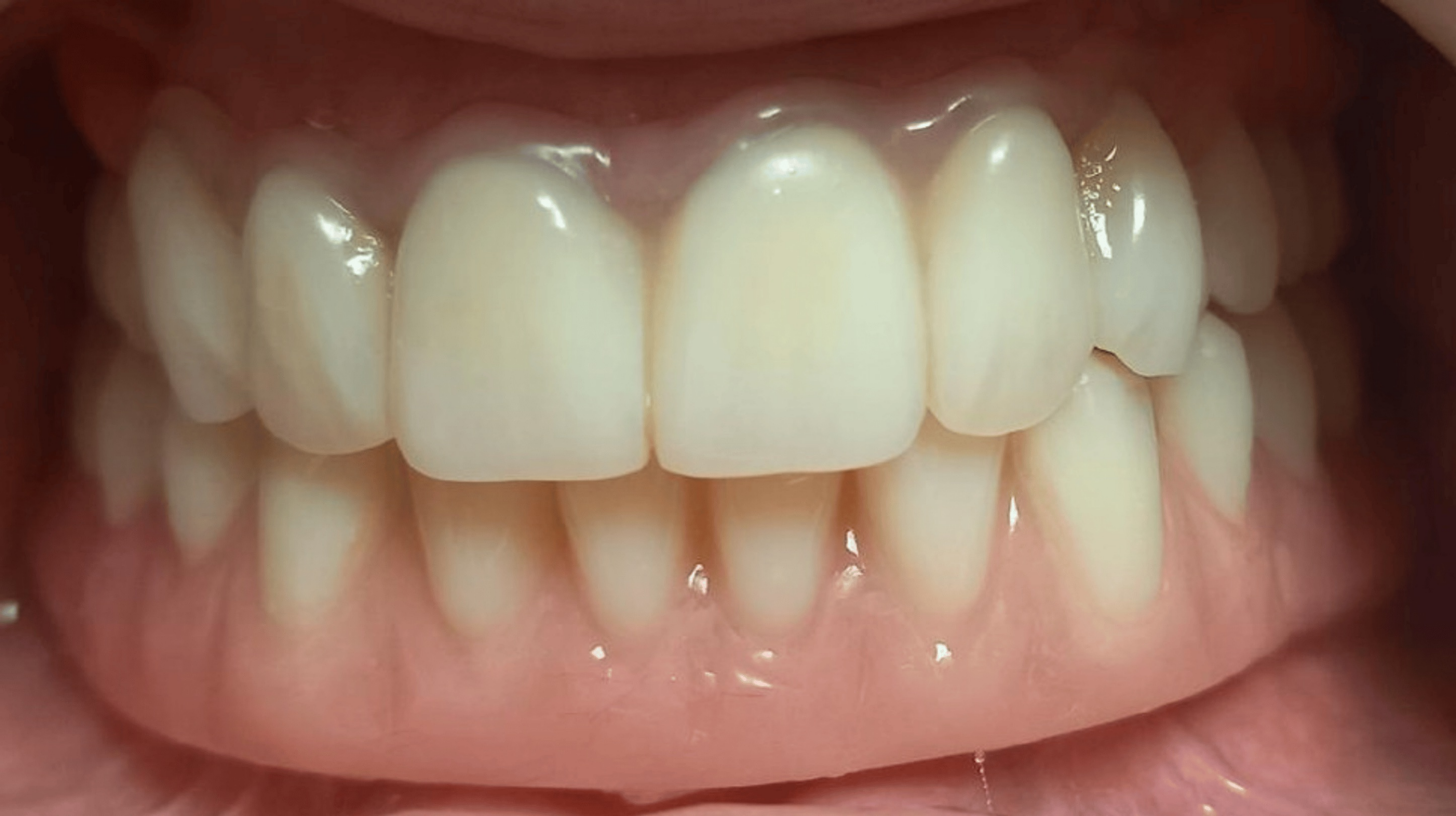
Final Upper Ivotion Lower Ivobase Dentures In Situ

At 18-month follow-up, the patient reported complete satisfaction with both function and aesthetics (Figure 12). Notably, nausea symptoms were no longer present during prosthesis use, suggesting successful neuromuscular adaptation. The patient resumed methotrexate therapy without complications. Table 2 contains a summary and timeline of patient management.

**Table 2:** Treatment Timeline

Time point	Clinical stage	Procedures / Events	Key Considerations
Initial presentation	Referral	Patient referred by maxillofacial surgeon with severely compromised dentition	Strong aesthetic demand; request for implants
Medical evaluation	Diagnosis and risk assessment	Psoriasis under methotrexate therapy; persistent vomiting and severe nausea	Implant placement contraindicated; limited treatment tolerance
Treatment planning phase	Interdisciplinary planning	Multiple consultations; consent obtained; communication with physician	Methotrexate temporarily discontinued to improve tolerance
Pre-prosthetic phase	Surgical intervention	Extraction of non-restorable teeth by maxillofacial surgeon	Healing period observed prior to prosthetic phase
Mandibular phase: step 1	Tooth preparation and impression	Preparation of mandibular abutments; gingival retraction; digital impressions with Trios 3	Digital workflow reduced nausea
Mandibular phase: step 2	Copings and framework	Fabrication and try-in of primary copings; border molding; polyether pickup impression	Verification of parallelism critical
Mandibular phase: step 3	Jaw relationship records and try-in	Occlusal rims; centric jaw relation; OVD established; denture teeth try-in (Phonares)	Aesthetic and phonetic validation
Mandibular delivery	Prosthesis placement	Ivobase fabrication; cementation of primary copings with RPD in situ	Achieved stable occlusion and aesthetics
Adaptation phase	Functional adaptation	Patient adapts to mandibular telescopic RPD	Improved confidence and compliance
Maxillary phase: step 1	Diagnostic and provisional	Digital scan; fabrication of flexible (Valplast-type) RPD	Used to determine functional borders due to nausea
Maxillary phase: step 2	Border adjustment	Progressive adjustment of palatal coverage	Full palate not tolerated; design individualized
Maxillary phase: step 3	Definitive planning	Tooth preparation; digital smile design; CAD/CAM framework and PMMA try-in	Patient approval of aesthetics
Maxillary delivery	Definitive prosthesis	Ivotion system fabrication; cobalt-chromium framework; cementation of primary copings with RPD in situ	Patient preference for conventional metal framework
Early post-delivery (0–2 days)	Complication	Decementation of primary copings due to excessive friction	Caused by high precision (0° taper) digital design
Complication management	Adjustment phase	Recementation; chairside adjustment of framework using rubber burs	Reduction of frictional forces
Short-term follow-up	Stabilization	Periodontal ligament adaptation and splinting effect achieved	No further decementation incidents
1 week-1 month	Early follow-up	Functional and aesthetic evaluation	Improved comfort and adaptation
6 months	Maintenance	Chairside relining (Quick Up) due to ridge resorption	Maintained prosthesis fit
12 months	Maintenance	Second relining procedure	Continued stability and comfort
18 months	Long-term outcome	Full functional rehabilitation achieved; no nausea	Patient fully satisfied; methotrexate resumed
Ongoing	Recall Program	Regular 6-month recall visits	Monitoring of abutments and prosthesis

## Discussion

A rigid double-crown telescopic system was selected for both arches. The primary copings were fabricated using a CAD/CAM workflow and designed as parallel-sided telescopic copings with a 0° taper (parallel-wall design) and a common path of insertion. This design was intentionally chosen to maximize frictional retention, enhance prosthesis stability, and promote favorable load distribution among the remaining abutment teeth.

The copings were prepared with adequate axial wall height (4-6 mm, in accordance with the available prosthetic space) to increase the contact surface area between the primary and secondary crowns. The secondary crowns were incorporated into a cobalt-chromium removable framework and engaged the primary copings through a friction-fit mechanism. Retention was achieved exclusively through the intimate contact of the 0° parallel surfaces, eliminating the need for visible clasps or additional attachment systems.

The rigid 0° telescopic design also provided cross-arch splinting of the abutment teeth, improved support and stability of the removable prostheses, and reduced prosthesis displacement during function. The enhanced stability was considered particularly beneficial in this patient, as minimizing prosthesis movement reduced intraoral stimulation and contributed to improved tolerance in the presence of severe gagging and medication-related nausea.

Parallelism was verified clinically and during laboratory procedures to ensure a single path of insertion and predictable retention. The high precision achieved through the CAD/CAM workflow resulted in exceptionally strong frictional retention; however, it also contributed to the early decementation events observed during the first week after delivery, which were subsequently resolved through recementation and selective adjustment of the secondary framework. Following recementation and minor chairside adjustment of the internal surfaces of the secondary framework, no additional decementation episodes occurred during the 18-month follow-up period.

The success of the rehabilitation was evaluated based on prosthesis survival, functional performance, patient satisfaction, aesthetic outcome, resolution of nausea symptoms, and absence of further biological or technical complications. The patient remained fully functional and satisfied throughout the follow-up period. Therefore, the decementation represented an early manageable complication rather than failure of the prosthetic concept.

The rehabilitation of partially edentulous patients with telescopic crown-retained removable prostheses remains a predictable and versatile treatment modality, particularly in medically compromised individuals. Long-term clinical studies have demonstrated favorable survival rates and acceptable maintenance requirements [11]. These outcomes highlight the reliability of telescopic systems as an alternative when implant therapy is limited or contraindicated.

A critical factor influencing the success of telescopic systems is their fabrication method. Conventional analog techniques rely heavily on laboratory craftsmanship, particularly in achieving optimal taper angles and frictional fit between primary and secondary crowns. Variations in manual procedures may lead to inconsistencies in retention and long-term performance [5]. In contrast, digital workflows utilizing CAD/CAM technologies offer enhanced standardization, allowing precise control over taper geometry and surface characteristics, which are key determinants of retention force. Traditional non-precious metal alloys continue to be among the most mechanically robust materials, with CAD/CAM-designed restorations demonstrating retention forces up to 40% greater than those produced by conventional casting methods [12].

A notable advancement in the domain of telescopic crowns pertains to material innovation, as clinicians endeavor to harmonize aesthetics, functionality, and biological compatibility. Material selection further differentiates digital and analog approaches. Traditional analogue systems commonly employ cast metal alloys, which have demonstrated durability but may be associated with extensive friction forces, increased wear, and aesthetic limitations [10]. Digital manufacturing has facilitated the introduction of high-performance polymers and ceramics, such as PEEK, PEKK, and zirconia, which exhibit favorable biomechanical properties, reduced weight, and improved aesthetics [13]. Additionally, studies have shown that CAD/CAM-fabricated telescopic crowns using these materials can achieve comparable or even superior retention stability over time [14,15].

Despite these advantages, digital workflows are not without limitations. Clinical constraints, such as restricted intraoral access or severe nausea--as observed in the present case--can complicate intraoral scanning procedures. While digital impressions are generally more comfortable for patients, their success is technique-sensitive, as they depict edentulous areas and consequently may require patient cooperation and operator experience. In such situations, modified analog techniques or hybrid workflows may provide a more practical solution [9].

From a biomechanical perspective, both analog and digital telescopic systems aim to achieve optimal load distribution along the abutment teeth. Finite element analyses have demonstrated that telescopic designs can effectively reduce stress concentrations compared with other attachment systems, thereby preserving supporting structures [16]. Furthermore, clinical evidence suggests that double crown-retained prostheses may result in comparable or even reduced abutment tooth loss compared to conventional clasp-retained removable partial dentures [17].

Patient-centered outcomes also play a significant role in treatment evaluation. Improvements in oral health-related quality of life, comfort, and aesthetics have been consistently reported with telescopic prostheses, regardless of fabrication method [18,19]. However, the precision and passive fit achievable with digital techniques may further enhance patient satisfaction by reducing complications related to misfit and wear.

Overall, while digital workflows offer clear advantages in terms of precision, material innovation, and reproducibility, analogue techniques remain clinically relevant, particularly in complex cases with anatomical or functional limitations. A combined or hybrid approach may therefore represent the most pragmatic strategy, allowing clinicians to tailor treatment to individual patient needs while leveraging the strengths of both methodologies.

According to House, the exacting patient is concerned about the appearance and efficiency of dentures, reluctant to accept the advice of the dentist, and opposed to the extraction of any remaining teeth. This category of patients is characterized by a highly critical, meticulous, and demanding attitude toward treatment, thereby necessitating extensive explanations, continuous reassurance, and meticulous clinician-patient communication throughout the management of the complex therapeutic procedure [20].

Limitations of the study and future directions

This report presents the management of a single medically compromised patient and, therefore, its findings should be interpreted with caution. As a case report, the study design does not permit generalization of the clinical outcomes to broader patient populations. Additionally, the follow-up period of 18 months, although sufficient to demonstrate short- and medium-term success, does not provide information regarding the long-term survival of the abutment teeth, maintenance requirements, or prosthesis longevity.

Another limitation is the absence of objective outcome measures, such as validated oral health-related quality-of-life questionnaires, patient satisfaction scales, masticatory performance assessments, or quantitative evaluation of nausea severity before and after treatment. Furthermore, no direct comparison was made between fully digital, conventional analogue, and hybrid workflows, limiting the ability to determine the relative benefits of each approach in patients with severe gag reflexes or medication-induced nausea.

Future studies should include prospective clinical investigations with larger cohorts of medically compromised patients to evaluate the long-term effectiveness and maintenance requirements of telescopic crown-retained removable prostheses. Comparative studies examining digital, analogue, and hybrid workflows are also warranted to identify the most effective strategies for managing patients with functional limitations such as severe nausea and exaggerated gag reflexes. In addition, further research should investigate patient-reported outcomes, objective functional parameters, and the influence of systemic diseases and immunomodulatory therapies on prosthetic adaptation and long-term treatment success. Such evidence would contribute to the development of evidence-based clinical protocols for the rehabilitation of complex medically compromised patients.

## Conclusions

Telescopic crown-retained removable prostheses are an effective option for medically compromised patients when implant therapy is contraindicated. This case highlights the importance of individualized planning to address systemic disease, medication side effects, and severe nausea. Digital workflows improve precision and comfort but require careful management to avoid friction-related complications. Long-term success depends on staged treatment, meticulous execution, patient adaptation, and regular maintenance.

## Appendices

Table 3 outlines the complete materials and workflow involved in the stepwise prosthodontic rehabilitation. 

**Table 3:** Complete materials and workflow for stepwise prosthodontic rehabilitation CAD/CAM: Computer-aided design and computer-aided manufacturing; PMMA: polymethyl methacrylate;

Step / procedure	Material / product	Company	Country	Purpose / clinical notes
Diagnostic radiographic assessment	NewTom GO 2D/3D	CEFLA S.C.	Italy	Panoramic imaging for diagnosis and treatment planning
Gingival retraction	Ultrapak retraction cord	Ultradent	USA	Exposure of preparation margins before digital impression
Digital impression acquisition	TRIOS 3 intraoral scanner	3Shape	Denmark	Digital scanning with reduced nausea stimulation
Preliminary impression	Alginate impression material	Kerr	USA	Initial impression for study cast and custom tray fabrication
Custom tray fabrication	Custom impression tray material ALMORE	ALMORE	Poland	Individual tray construction
Border molding	Greenstick compound	Kerr	USA	Functional molding of tray peripheries
Definitive pickup impression	Polyether impregum	3M ESPE	Germany/USA	Accurate transfer of copings and soft tissue detail
Primary telescopic copings	CAD/CAM metal copings	Laboratory fabricated	—	Primary retainers cemented to prepared abutments
Secondary telescopic framework	Vitalium 2000 cobalt–chromium alloy framework	Dentsply Sirona	USA/Germany	Rigid secondary structure for removable prosthesis
Occlusal registration	Wax rims	Generic	—	Recording vertical dimension and centric relation
Jaw relation record	BMS modeling wax	BMS Dental	Italy	Stabilization and recording of jaw relationship
Denture teeth setup	Phonares	Ivoclar Vivadent	Liechtenstein	Functional and esthetic tooth arrangement
Mandibular denture processing	Ivobase injection system	Ivoclar Vivadent	Liechtenstein	Injection molding of definitive prosthesis
Cementation of copings	Ketac Cem glass ionomer cement	3M	USA	Definitive luting of primary copings
Digital prosthesis design	DentalCAD 3.2	exocad GmbH	Germany	CAD design of telescopic restorations
Esthetic-functional try-in	Telio CAD (PMMA)	Ivoclar	Liechtenstein	Mock-up verification of esthetics and function
Maxillary definitive acrylic fabrication	Ivotion	Ivoclar Vivadent	Liechtenstein	CAD/CAM denture base fabrication
Chairside friction adjustment	Dedeco rubber polishers	Dedeco International	USA	Reduction of excessive frictional retention
Hard denture relining	Quick up	VOCO	Germany	Compensation for post-extraction ridge resorption
Chemical plaque control	0.12% chlorhexidine mouthrinse		—	Monthly antimicrobial maintenance
Daily preventive hygiene	Sodium fluoride toothpaste		—	Caries prevention for abutment protection
